# New and Emerging Therapies for Osteoporosis

**DOI:** 10.4061/2010/318320

**Published:** 2010-12-26

**Authors:** E. Michael Lewiecki, Manuel Diaz Curiel, Joao Lindolfo Borges, Annie Kung, Maria Luisa Brandi, Hans Peter Dimai

**Affiliations:** ^1^New Mexico Clinical Research & Osteoporosis Center, 300 Oak St. NE, Albuquerque, NM 87106, USA; ^2^Jimenez Diaz Fundacion, Avenida Reyes Catolicos 2, Madrid 28040, Spain; ^3^Universidade Catolica de Brasilia, DF, Brazil; ^4^University of Hong Kong, 102 Pokfulam Road, Hong Kong; ^5^Maria Luisa Brandi, University of Florence, Viale Pieraccini 6, Florence 50139, Italy; ^6^Medical University Graz, Auenbruggerplatz 15, Graz A-8036, Austria

Osteoporosis is a common skeletal disease that increases the risk of fracture, with serious clinical and economic consequences. It can be diagnosed by dual-energy X-ray absorptiometry (DXA), even before a fracture has occurred, using the World Health Organization (WHO) criteria according to the patient's T-score. The WHO has also developed a fracture risk assessment tool (FRAX) to estimate the 10-year probability of major osteoporotic fracture (clinical spine, hip, proximal humerus, and distal forearm) and hip fracture, using clinical risk factors for fracture and femoral neck bone mineral density (BMD), if available. Cost-effective pharmacological agents that have been proven to reduce fracture risk in patients at high risk for fracture are now widely available. However, despite great progress in the management of osteoporosis, it remains a disease that is underrecognized and undertreated; if treatment is started, persistence is often poor, with only about 50% of patients who are prescribed medication for osteoporosis still taking it 1 year later. Even when treatment is taken correctly and for a sufficient length of time for the patient to benefit from reduction in fracture risk, there may nevertheless be limitations in effectiveness (note the lack of evidence for reduction in the risk of hip fractures or other nonvertebral fractures with some agents), limitations in the duration of therapy (e.g., no more than 24 months of lifetime teriparatide in the US), and concerns regarding long-term safety, such as atypical femur fractures and osteonecrosis of the jaw with bisphosphonates. For all of these reasons, the goal of reducing the global burden of osteoporotic fractures is not being fully achieved. 

This special issue of the *Journal of Osteoporosis* describes new and emerging approaches to treatment that offer the potential to reduce the risk of fractures or manage their consequences better than what is currently observed in clinical practice. In recent years, our understanding of the pathophysiology of osteoporosis and the regulation of bone remodeling at the molecular level have undergone tremendous advances, leading to the investigation of drugs that target specific molecules in order to modulate the bone remodeling process. For example, the discovery that receptor activator of nuclear factor kappa B ligand (RANKL) is the principal regulator of osteoclastic bone resorption led to the development of denosumab, a fully human monoclonal antibody to RANKL. This potent antiresorptive agent, administered as a 60 mg subcutaneous injection every 6 months, recently received regulatory approval for the treatment of women with postmenopausal osteoporosis (PMO) at high risk for fracture. It has been shown to increase BMD, reduce bone turnover marker levels, and reduce the risk of vertebral fractures, hip fractures, and nonvertebral fractures in women with PMO.

Wnt signaling initiated by the binding of Wnt proteins to the low density lipoprotein-related protein (LRP5/6)-frizzled receptor complex has recently been recognized as an important upregulator of osteoblastic bone formation; sclerostin and Dickkopf-1 (DKK-1) are natural inhibitors of Wnt signaling. In this issue, J. J. Mason and B. O. Williams describe a rare genetic disorder, sclerosteosis, resulting from a mutation of the *SOST* gene that encodes for sclerostin, and van Buchem disease, a related disorder caused by a mutation closely linked to *SOST* on chromosome 17q11.2. Patients with sclerosteosis and van Buchem disease have high bone mass due to downregulation of sclerostin, suggesting that a therapeutic agent that downregulates sclerostin in a controllable fashion might be a potent osteoanabolic treatment for patients with osteoporosis. Mason and Williams review many of the studies that have enhanced our understanding of the regulators of Wnt signaling and lead to the investigation of compounds with potential therapeutic applications through their effects on sclerostin or DKK1. A fascinating new finding, yet to be fully elucidated, is that serotonin produced by enterochromaffin cells in the duodenum also downregulates Wnt signaling, raising the possibility that modulation of serotonin production or activity might also be an effective treatment for patients with osteoporosis. In a related paper by S. Silverman in this issue, the preclinical and clinical studies of sclerostin inhibition are presented. 

The drugs used to treat osteoporosis are generally considered to be in 1 of 2 categories—antiresorptive (e.g, bisphosphonates) or osteoanabolic (e.g., teriparatide). Interestingly, some drugs may “uncouple,” at least in part, the closely related processes of bone resorption and formation. Strontium ranelate may be such a drug. Another, perhaps, is odanacatib, an investigational agent that inhibits cathepsin K, a protease produced by osteoclasts that is largely responsible for the degradation of the bone collagen matrix. J. L. Perez-Castrillon et al. review what is now known about the role of cathepsin K in health and disease, followed by data from phase 1 and phase 2 clinical trials with odanacatib. This drug is currently under investigation in a large phase 3 clinical trial to evaluate antifracture efficacy in women with PMO. Other papers in this issue cover new developments concerning skeletal heath in areas as diverse as bisphosphonate nanoparticles, melatonin, and thalassemia.

The papers in the issue were selected from many excellent submissions. We give our thanks to all authors of these submissions and to the numerous reviewers who kindly donated their time and expertise in helping select and revise those published here. 

**E. Michael Lewiecki** **E. Michael Lewiecki** **Manuel Diaz Curiel** **Manuel Diaz Curiel** **Joao Lindolfo Borges** **Joao Lindolfo Borges** **Annie Kung** **Annie Kung** **Maria Luisa Brandi** **Maria Luisa Brandi** **Hans Peter Dimai** **Hans Peter Dimai**

