# Corrigendum to “New and Emerging Therapies for Osteoporosis”

**DOI:** 10.1155/2017/4175180

**Published:** 2017-06-28

**Authors:** E. Michael Lewiecki, Manuel Diaz Curiel, Joao Lindolfo Borges, Annie Kung, Maria Luisa Brandi, Hans Peter Dimai

**Affiliations:** ^1^New Mexico Clinical Research & Osteoporosis Center, 300 Oak St. NE, Albuquerque, NM 87106, USA; ^2^Jimenez Diaz Fundacion, Avenida Reyes Catolicos 2, Madrid 28040, Spain; ^3^Universidade Catolica de Brasilia, DF, Brazil; ^4^University of Hong Kong, 102 Pokfulam Road, Hong Kong; ^5^Maria Luisa Brandi, University of Florence, Viale Pieraccini 6, Florence 50139, Italy; ^6^Medical University Graz, Auenbruggerplatz 15, Graz A-8036, Austria

In the article titled “New and Emerging Therapies for Osteoporosis” [1], there was an error regarding the FRAX® tool, which should be clarified as follows.

The article notes: “The WHO has also developed a fracture risk assessment tool (FRAX) to estimate the 10-year probability of major osteoporotic fracture (clinical spine, hip, proximal humerus, and distal forearm) and hip fracture, using clinical risk factors for fracture and femoral neck bone mineral density (BMD), if available.” However, the World Health Organization (WHO) did not develop, test, or endorse the FRAX tool or its recommendations [2]. The metabolic bone disease unit at the University of Sheffield that developed FRAX was a WHO Collaborating Centre from 1991 to 2010, but treatment guidelines must undergo a formal process before they can be endorsed by the WHO.
